# Use of Renal Replacement Therapy May Influence Graft Outcomes following Liver Transplantation for Acute Liver Failure: A Propensity-Score Matched Population-Based Retrospective Cohort Study

**DOI:** 10.1371/journal.pone.0148782

**Published:** 2016-03-01

**Authors:** Stephen R. Knight, Gabriel C. Oniscu, Luke Devey, Kenneth J. Simpson, Stephen J. Wigmore, Ewen M. Harrison

**Affiliations:** 1 Scottish Liver Transplant Unit, Royal Infirmary of Edinburgh, Edinburgh, EH16 4SA, United Kingdom; 2 Pipeline Futures Group, GSK, 1250 South Collegeville Rd, Collegeville, Pennsylvania, 19426, United States of America; Ohio State University College of Medicine, UNITED STATES

## Abstract

**Introduction:**

Acute kidney injury is associated with a poor prognosis in acute liver failure but little is known of outcomes in patients undergoing transplantation for acute liver failure who require renal replacement therapy.

**Methods:**

A retrospective analysis of the United Kingdom Transplant Registry was performed (1 January 2001–31 December 2011) with patient and graft survival determined using Kaplan-Meier methods. Cox proportional hazards models were used together with propensity-score based full matching on renal replacement therapy use.

**Results:**

Three-year patient and graft survival for patients receiving renal replacement therapy were 77.7% and 72.6% compared with 85.1% and 79.4% for those not requiring renal replacement therapy (P<0.001 and P = 0.009 respectively, n = 725). In a Cox proportional hazards model, renal replacement therapy was a predictor of both patient death (hazard ratio (HR) 1.59, 95% CI 1.01–2.50, P = 0.044) but not graft loss (HR 1.39, 95% CI 0.92–2.10, P = 0.114). In groups fully matched on baseline covariates, those not receiving renal replacement therapy with a serum creatinine greater than 175μmol/L had a significantly worse risk of graft failure than those receiving renal replacement therapy.

**Conclusion:**

In patients being transplanted for acute liver failure, use of renal replacement therapy is a strong predictor of patient death and graft loss. Those not receiving renal replacement therapy with an elevated serum creatinine may be at greater risk of early graft failure than those receiving renal replacement therapy. A low threshold for instituting renal replacement therapy may therefore be beneficial.

## Introduction

The management of acute liver failure (ALF) has been transformed by the introduction of liver transplantation. ALF is associated with significant morbidity and mortality, with outcome dependent on several factors [1]. The King’s College Hospital Criteria^2^ were developed to predict survival and identify individuals requiring transplantation in whom spontaneous recovery is unlikely. Elevated serum creatinine, a marker of renal impairment, is known to be a predictor of poorer patient survival and is included as a factor for acetaminophen-induced ALF in the King’s college criteria [2].

Furthermore, renal impairment is associated with reduced survival in patients undergoing both elective and urgent orthotopic liver transplants [3]. Estimates suggest around 20% of patients with chronic liver failure develop renal dysfunction [4]. In acute liver failure renal dysfunction occurs in up to two-thirds of patients [5], with up to half requiring renal replacement therapy before transplantation [6].

Elevated pre-operative serum creatinine levels are associated with increased risk of post-operative sepsis [7–9], the requirement for post-operative dialysis [9, 10] and short-term graft and patient survival [3, 11] in orthotopic liver transplantation.

However, the relationship between renal impairment, renal replacement therapy (RRT) and patient and graft survival following liver transplantation in ALF remains unclear. A single-centre study suggested pre-operative renal dysfunction significantly reduces patient survival [5] but it is not known if reduction in elevated serum creatinine through RRT increases long-term patient and graft survival post-operatively.

Using a national database covering a 10-year period, we aimed to perform a population-based cohort study comparing patients undergoing liver transplantation for acute liver failure with and without a requirement for RRT. To minimise confounding factors as far as is possible, we used propensity-score based matching to balance treatment groups.

## Materials and Methods

Data were extracted from the United Kingdom Transplant Registry (UKTR), a mandatory registry held by National Health Service (NHS) Blood and Transplant on 16^th^ August 2012 for the period 1 January 2001 and 31 December 2011. Permission was provided by NHS Blood and Transplant to explore outcomes following donation after cardiac (DCD) and brain death (DBD) liver transplantation. This study used only anonymised data obtained as part of usual care and thus did not need NHS ethical review under the terms of the Governance Arrangements for Research Ethics Committees (A Harmonised Edition) [12]. All data was anonymised by NHS Blood and Transplant.

Data included all first-time deceased donor liver-only transplant patients receiving liver transplantation for ALF (United Kingdom (UK) Transplant Super Urgent Scheme Category 1–10; S1 Table). In the UK, patients with ALF who are predicted by the King’s college criteria [2] as unlikely to spontaneously recover liver function and meet psychological and other criteria are eligible for transplantation, receiving national priority for any donor that becomes available. Patients do not have chronic liver disease, except in the circumstance of re-transplantation for hepatic artery thrombosis which was excluded in this analysis. Graft survival was defined as all-cause graft loss including graft failure or patient death, whichever came first. In addition, data did not include patients less than 18 years old, split or reduced liver transplants, multi-organ transplants or heterotopic transplants.

Data were collected for pre-operative recipient characteristics immediately prior to transplantation: age, gender, race, serum creatinine, urea, bilirubin, albumin, sodium, potassium, haemoglobin, white blood cell count, platelet count, aetiology of liver failure, encephalopathy grade, intracranial pressure monitoring status, presence of sepsis, life-style activity score (S2 Table), ventilatory support status and the use of renal replacement therapy (both haemodialysis and filtration). Within the UK indication for commencing RRT is centre-specific, however national guidelines suggest its use in the presence of refractory hyperkalaemia/electrolyte abnormalities, acute kidney injury with severe organ failure, refractory pulmonary oedema and oliguria (<0.3 ml/kg/hr for 24 hours) [13]. Liver failure aetiology was determined according to the classification system described by Gotthardt *et al* for acute liver failure [14]: viral, drug toxicity, metabolic, vascular, and miscellaneous aetiology.

Data were collected for donor characteristics: age, gender, weight, height, race, cause of death, donor type (DCD/DBD), presence of steatosis, and cold ischaemic time; together with operative characteristics: organ anatomy, operative reperfusion time and number of blood units used intra-operatively.

Kaplan-Meier survival methods with a log-rank test were used to identify pre-operative factors which influenced graft and patient survival. Hazard ratios and confidence intervals (CI) were determined for patient and graft survival using Cox proportional hazards model (CPH). In univariable analyses, continuous variables were dichotomized according to mean values, while in multivariable models these were treated as continuous: for example, age (<40 vs. ≥40 years), creatinine (<120 vs. ≥120 μmol/L), urea (<5 vs. ≥5 μmol/L) and haemoglobin (<10 vs. ≥10 g/dL). A CPH model was used to determine independent risk factors predicting survival following transplantation for ALF using variables found to be significant in univariable analysis. When performing the analysis, creatinine was centred at 90 μmol/L for RRT in order to provide interpretation at a clinically relevant creatinine concentration The underlying hazard function was assessed and seen to be constant, and no time dependent variables were specified. Calibration of the final CPH model with actual patient survival was checked and found to be reasonable (S1 Fig). We explored applying a cubic spline to the continuous variables in order to enhance calibration, however this did not significantly improve model fit.

The measures above go some way to controlling for differences in characteristics between treatment groups. However, it is acknowledged that significant selection bias exists in assigning patients to one treatment or the other [15]. In order to try and reduce the influence of selection bias as far as is possible, propensity-score matching was performed using all available recipient variables: gender, age, ethnicity, cause of liver failure, life-style activity score, ventilation status, encephalopathy grade, intracranial pressure monitoring status, sepsis status, haemoglobin, white cell count, platelet count, albumin, bilirubin and sodium. Logistic regression was used to determine the probability of treatment group membership, which was used for matching. “Nearest neighbour” matching is often utilised, however, with these data there is an excess of patients in the treated group compared to the control. Covariate balance is not possible using this method. “Full matching” is used in which a fully matched sample is composed of matched sets, where each matched set contains one treated unit and one or more controls (or one control unit and one or more treated units) [16].

Full matching is optimal in minimizing a weighted average of the estimated distance measure between each treated subject and each control subject within each subclass. This can be thought of as “weighting” the control group to look as similar to the treated group as possible. Thus, as an example, to optimally match a single patient in the treated group, it may be best to use two patients from the control group with each given a weighting of 0.5. The distribution of the propensity scores in treated and control groups was checked (S2 Fig) and covariate balance determined (S3 Table). Cox proportional hazards models were constructed using the matched dataset and used to identify factors predicting patient and graft survival, including graphical representations. The matching and weighting are accounted for in the analysis by specifying the subclass as a cluster, thus ensuring robust estimation of the variance, and adjusting the contribution of each patient to the analysis using the weight determined above [17].

All statistical analysis was performed using R 2.15.3 (R Foundation for Statistical Computing) with Zelig [18], survival, epitools, rms, Match-It and optmatch packages. Statistical significance is two-sided and data are presented as mean with bootstrapped 95% CI, unless stated otherwise.

## Results

### Patient characteristics

There were a total of 5753 liver transplants during the study period, with 725 performed for ALF. The median age of patients receiving a liver transplant for ALF was 38 (interquartile range (IQR) 28 to 49) years, with a greater proportion received by females and Caucasians (Table 1). When aetiology was classified according to Gotthardt *et al* [13], 299 (41.2%) patients presented with ALF secondary to drug-toxicity, a metabolic cause in 40 (5.5%), viral in 36 (5.0%) and vascular in 22 (3.0%), with miscellaneous accounting for 328 (45.3%). Immediately prior to transplantation, the median value of serum creatinine was 128 μmol/L (IQR 90 to 187), while more than half (389/725) received renal replacement therapy. Within this group, 357 (91.8%) received filtration while 32 (8.2%) received haemodialysis. No patients had significant chronic kidney disease prior to acute liver failure. Donors had a median age of 45 (IQR 33 to 56) with the majority of organs from donors with brain death (719 patients, 99.2%).

**Table 1:** Characteristics of patients, donors and grafts for those receiving liver transplantation for acute liver failure. Values expressed as median (interquartile range) and number (percent) unless otherwise stated. Missing data for each characteristic is reported, with absolute number in each group included in parentheses. ALF, acute liver failure; SD, standard deviation; INR, international normalised ratio; RRT, renal replacement therapy; ICH/CVA/Hypoxic, intracranial haemorrhage/cerebral vascular accident/hypoxic brain injury; ICU, intensive care unit; DCD, donation after cardiac death.

	No RRT (N = 336)	RRT (N = 389)	Total (N = 725)	Missing data
**Recipient characteristics**				
Age, years	40 (31–51)	36 (27–45)	38 (28–49)	0
Male:Female	1:2.4	1:1.5	1:1.9	0
Ethnicity, Caucasian	265 (78.9)	338 (86.9)	603 (83.2)	0
1^st^ transplant	336 (100.0)	389 (100.0)	725 (100.0)	0
**Aetiology of ALF**				
Viral	22 (6.5)	14 (3.6)	36 (5.0)	0
Drug toxicity	61 (18.2)	238 (61.2)	299 (41.2)	0
Vascular	11 (3.3)	11 (2.8)	22 (3.0)	0
Metabolic	28 (8.3)	12 (3.1)	40 (5.5)	0
Miscellaneous	214 (63.7)	114 (29.3)	328 (45.3)	0
**Characteristics immediately prior to transplant**				
Encephalopathy, mean grade (SD)	3.6 (1.5)	4.5 (1.4)	4.1 (1.2)	3 (1/2)
Sepsis	21 (6.2)	35 (9.0)	56 (7.7)	7 (4/3)
Mechanical ventilation	142 (42.3)	353 (90.8)	495 (68.3)	0
Intracranial pressure measurement				
Normal	16 (4.8)	50 (12.9)	69 (7.5)	0
Raised	16 (4.8)	117 (30.1)	136 (14.7)	0
Not used	304 (90.5)	222 (57.1)	526 (72.6)	0
Bilirubin, μmol/L	337 (184–462)	147 (86–296)	235 (106–404)	1 (0/1)
Albumin, g/L	25 (21–28)	24 (20–28)	24. (21–28)	3 (2/1)
Sodium, mmol/L	139 (134–144)	141 (136–146)	140 (135–145)	0
Creatinine, μmol/L	99 (75–135)	157 (117–237)	128 (90–187)	0
Urea, μmol/L	4.1 (2.3–7.3)	5.5 (3.3–8.7)	4.9 (2.7–8.2)	9 (2/7)
INR	2.7 (2.0–4.3)	3.1 (2.1–5.3)	2.9 (2.1–4.8)	49 (13/36)
Haemoglobin, g/dL	10.6 (9.0–12.4)	9.0 (8.1–10.3)	9.6 (8.4–11.5)	0
Platelets, 10^9^/L	110 (74–168)	70 (47–102)	86 (56–134)	0
pH	7.45 (7.40–7.47)	7.35 (7.23–7.43)	7.40 (7.30–7.46)	582 (280/302)
**Donor/graft characteristics**				
Age, years	45 (33–55)	45 (33–56)	45 (33–56)	0
Weight, kg	70 (62–80)	70 (65–80)	70 (65–80)	1 (1/0)
Height, cm	168 (160–177)	170 (163–178)	170 (162–178)	10 (5/5)
**Cause of death**				
ICH/CVA/Hypoxic	261 (77.7)	283 (72.7)	544 (75.0)	0
Trauma	47 (14.0)	68 (17.5)	115 (15.9)	0
Other	28 (8.3)	38 (9.8)	66 (9.1)	0
ICU days	1.7 (1.3–3.3)	1.8 (1.3–2.9)	1.8 (1.3–3.1)	51 (25/26)
Ethnicity, Caucasian	326 (97.0)	376 (96.7)	702 (96.8)	3 (3/0)
Steatosis	108 (32.1)	138 (35.5)	246 (33.9)	20 (13/7)
DCD proportion	0	6 (1.5)	6 (0.8)	0
Cold ischemic time, min	567 (474–671)	537 (437–640)	549 (451–654)	26 (6/20)

### Patient and graft survival following liver transplantation for ALF

In univariable analyses, the hazard of death for those on RRT was almost twice that of those not on RRT (Hazard ratio 1.77, 1.28 to 2.44, P<0.001; Table 2). Other factors significantly associated with reduced patient survival at 3 years were recipient age >40 years old (Hazard ratio 1.71, 95% CI 1.25 to 2.33, P<0.001), pre-operative serum creatinine ≥120 μmol/L (1.93, 1.40 to 2.68, P<0.001), the use of mechanical ventilation (2.00, 1.37 to 2.94, P<0.001). In those patients with a pre-operative haemoglobin greater than 10 g/dL, patient death was significantly reduced (0.72, 0.53 to 0.99, P = 0.042) while gender, acetaminophen-induced ALF and sepsis had no impact. All other variables (from Table 1) were tested and none found to be associated with patient survival, including donor age, donor/recipient ethnicity, and cold ischaemic time (data not shown). Furthermore, the modality of RRT (either filtration or haemodialysis) did not impact upon either patient or graft survival (data not shown).

**Table 2:** Univariable analysis of risk factors for patient death and graft loss after liver transplantation for acute liver failure. Hazard ratios were determined using Cox Proportional Hazards model. Values in parentheses are 95% confidence intervals.

	Patient death		Graft loss	
	Hazard ratio	P-value	Hazard ratio	P-value
Recipient age (≥40)	1.71(1.25–2.33)	<0.001	1.39 (1.05–1.84)	0.024
Gender (female)	1.00 (0.73–1.39)	0.984	1.03 (0.77–1.39)	0.833
Acetaminophen-induced ALF	1.20 (0.87–1.66)	0.267	0.84 (0.62–1.13)	0.246
Haemoglobin (≥10 g/dL)	0.72 (0.53–0.99)	0.042	0.88 (0.81–0.94)	<0.001
Creatinine (≥120 μmol/L)	1.93 (1.40–2.68)	<0.001	1.76 (1.31–2.36)	<0.001
RRT	1.77 (1.28–2.44)	<0.001	1.47 (1.10–1.97)	0.009
Ventilatory support	2.00 (1.37–2.94)	<0.001	1.71 (1.22–2.40)	0.002
Sepsis	1.46 (0.87–2.44)	0.153	1.49 (0.93–2.40)	0.097

**Fig 1:**
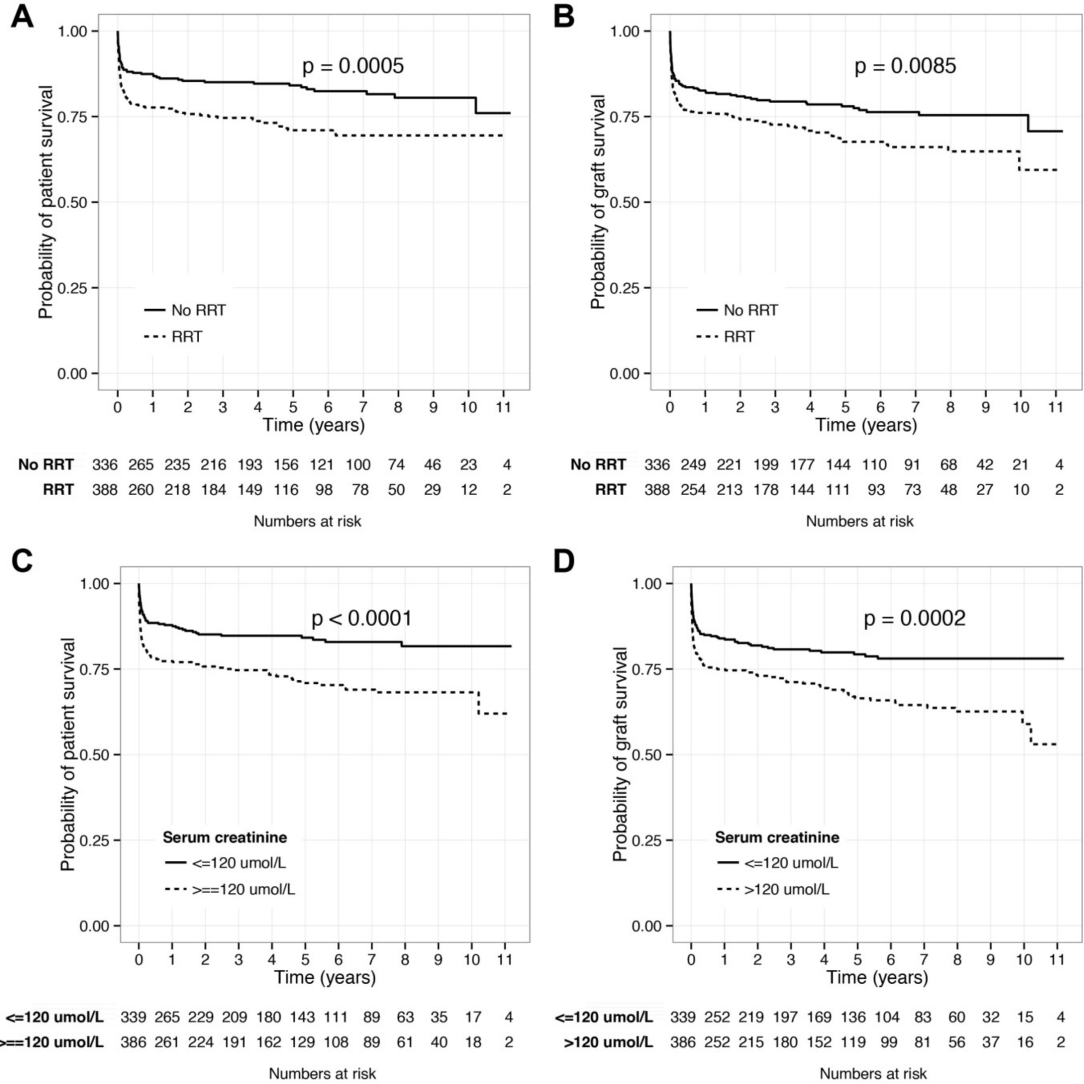
The effect of renal replacement therapy and serum creatinine on survival. Kaplan-Meier plots demonstrating the effect of both renal replacement therapy (A&B) and pre-operative serum creatinine (C&D) on patient and graft survival respectively in liver transplantation for acute liver failure. P value calculated using log-rank test.

The probability of patient survival at 1 and 3 years were 77.7% (95% CI 73.6–81.9) and 74.6% (70.3–79.2) in patients receiving RRT compared with 87.5% (84.0–91.1) and 85.1% (81.3–89.0) in those not requiring RRT (log-rank test, P = 0.0005; Fig 1A).

Univariable analysis demonstrated predictors of graft failure to be use of RRT (Hazard ratio 1.47, 1.10 to 1.97, P = 0.009), recipient age (1.39, 95% CI 1.05 to 1.84, P = 0.024), pre-operative serum creatinine concentration (1.76, 1.31 to 2.36, P<0.001), and the use of mechanical ventilation (1.71, 1.22 to 2.40, P = 0.002; Table 2). A pre-operative haemoglobin concentration greater than 10 g/dL was associated with a higher graft survival rate at 3 years post transplantation (0.88, 0.81 to 0.94, P<0.001).

Graft survival probabilities show a similar pattern: at 1 and 3 years these were 76.1% (95% CI 72.0 to 80.5) and 72.6% (68.2 to 77.4) in patients receiving RRT compared with 82.6% (78.6 to 86.8) and 79.4% (75.1 to 84.0) in those not requiring RRT (log-rank test, P = 0.0085; Fig 1B). Primary non-function (13 episodes, 6.9%), chronic rejection (13, 6.9%) or hepatic artery thrombosis (13, 6.9%, S4 Table) were the predominant causes of graft failure in the cohort. Graft failure was the cause of patient death in 3 cases.

A total of 161 (22.2%) patients in the cohort died. Causes of death included multi-organ failure (54 cases, 33.5%), septicaemia (22, 13.7%), cardiac causes (5, 3.1%), major haemorrhage (5, 3.1%) and cerebrovascular accidents (5, 3.1%).

### Relationship between serum creatinine, RRT and survival

Elevated pre-operative serum creatinine was found to be significantly associated with a reduction in both patient and graft survival in univariable analyses (Fig 1C and 1D). At 1 year, patient and graft survival were 87.8% (95% CI 84.4–91.4) and 84.0% (95% CI 80.1–88.0) in patients with a baseline creatinine of <120 μmol/L. Patients with a higher creatinine concentration (≥120 μmol/L) had a significantly worse outcome for both patient (77.3% (95% CI 73.2–81.6; log-rank test, P<0.0001)) and graft survival (74.9% (95% CI 70.7–79.4; log-rank test, P = 0.0002)).

In multivariable Cox proportional hazards (CPH) models, use of RRT was associated with patient death (hazard ratio (HR) 1.59, 95% CI 1.01 to 2.50, p = 0.044; Table 3) when creatinine was centred at a value of 90 μmol/L. Other variables associated with patient death were recipient age ((per 10 years) 1.32, 1.17 to 1.49, P<0.001) and the use of ventilatory support (1.69, 1.09 to 2.63, P = 0.020). A pre-operative haemoglobin greater than 10 g/dL was associated with a reduced hazard of patient death (0.90, 0.83 to 0.98, P = 0.018). However, RRT was not a predictor of graft loss (1.39, 0.92 to 2.10, P = 0.114), while pre-operative haemoglobin greater than 10g/dL was independently associated with graft loss (0.92, 0.85 to 0.99, P = 0.025). A total of 9 patients were excluded from the multivariate analysis due to missing values.

**Table 3:** Multivariable analysis of pre-operative risk factors for patient death and graft loss in those undergoing liver transplantation for acute liver failure. Cox Proportional Hazards model using variables found to be significant in univariable analysis (P<0.05) and those thought to be clinically significant. Serum creatinine was centred at a value of 90 μmol/L for the purposes of the analysis. Patient survival data was only considered for patients receiving their first liver transplantation. Values in parentheses are 95% confidence intervals.

	Patient death		Graft loss	
	Hazard Ratio	P-value	Hazard Ratio	P-value
Recipient age (/10 years)	1.32 (1.17–1.49)	<0.001	1.17 (1.04–1.31)	0.007
Haemoglobin	0.90 (0.83–0.98)	0.018	0.92 (0.85–0.99)	0.025
Creatinine (/10 μmol/L)	1.04 (1.01–1.07)	0.008	1.05 (1.02–1.07)	0.001
RRT	1.59 (1.01–2.50)	0.044	1.39 (0.92–2.10)	0.114
Ventilatory support	1.69 (1.09–2.63)	0.020	1.47 (0.92–2.10)	0.057
Sepsis	1.17 (0.69–1.98)	0.558	1.30 (0.80–2.10)	0.290
Creatinine:RRT interaction	0.96 (0.93–0.99)	0.038	0.96 (0.93–0.99)	0.009

The influence of serum creatinine concentration on outcome was examined accounting for an expected interaction with RRT, that is, the strength of association of serum creatinine with a given outcome might be expected to be different for those on RRT compared with those not on RRT. Serum creatinine was seen to be an independent predictor of both patient death (HR (per 10 units) 1.04, 1.01 to 1.07, P = 0.008) and graft loss (1.05, 95% CI 1.02 to 1.07, P = 0.001) even after accounting for the interaction with RRT.

**Fig 2:**
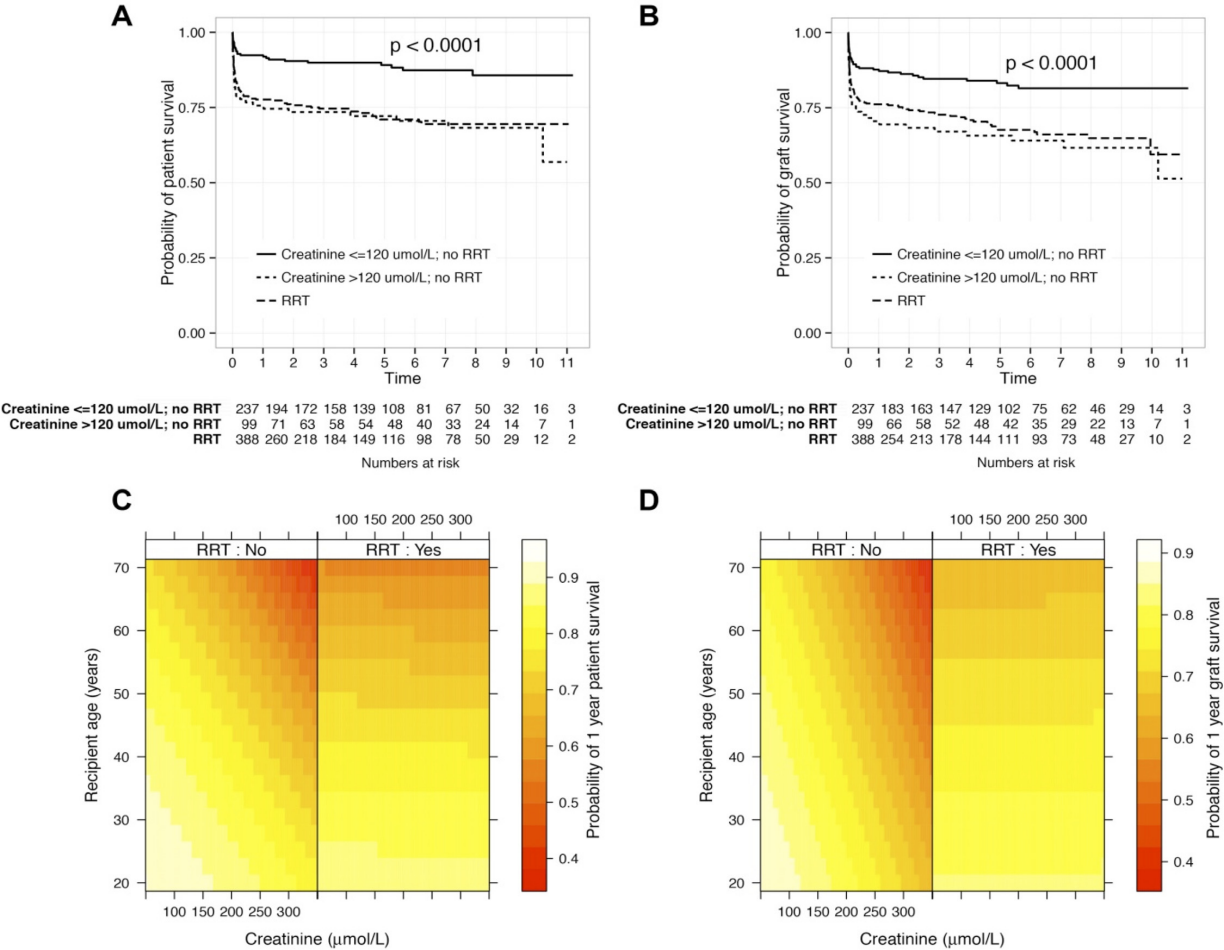
The interaction between renal replacement therapy and elevated creatinine on patient and graft survival. Kaplan-Meier plots demonstrating the interaction between pre-operative creatinine concentration and requirement for RRT on patient (A) and graft survival (B); P value calculated using log-rank test. Probability of death at one year following liver transplant by pre-operative serum creatinine level, RRT and recipient age is shown for patient (C) and graft survival (D). Models use Cox proportional hazards, with co-variable patient characteristics adjusted to haemoglobin concentration of 10 g/dL, requirement of mechanical ventilation and absence of sepsis.

The relationship between pre-operative creatinine, use of RRT and outcome was explored with modelling (Fig 2). Patients with a pre-operative serum creatinine ≥120 μmol/L in the absence of RRT had a similar probability of both patient death and graft failure as those receiving RRT (Fig 2A and 2B). Using a two-dimensional heat-map, the relationship between age, serum creatinine, use of RRT and survival is demonstrated (Fig 2C and 2D). These plots show that those with an elevated creatinine but not on RRT have a numerically higher probability of both death and graft failure than those on RRT.

### Propensity-score based matched analysis

**Fig 3:**
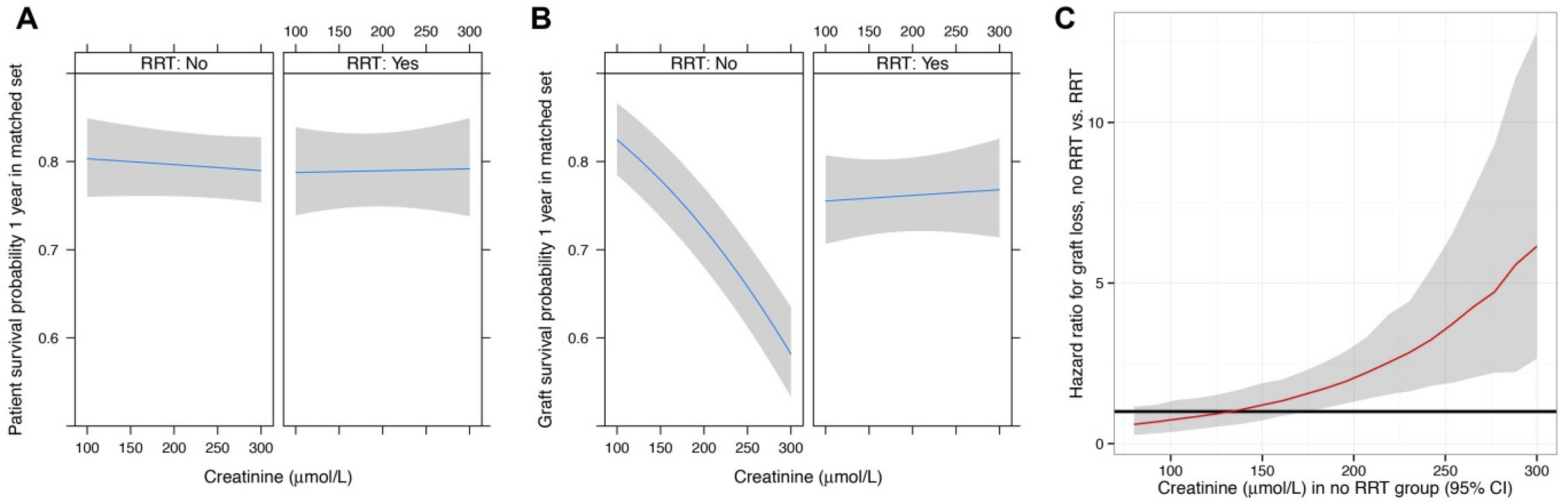
Renal replacement therapy and survival in a matched dataset. Probability of patient (A) and graft survival (B) at one year following liver transplant by pre-operative serum creatinine level and RRT requirement in a matched dataset. In patients not receiving RRT, a pre-operative serum creatinine of greater than 175 μmol/L had a significantly greater risk of graft failure compared to those receiving RRT (C; black line indicates hazard ratio = 1, shaded areas represent 95% CI). Cox proportional hazards model with groups fully matched for baseline covariates used for each analysis.

The interaction between pre-operative serum creatinine and the requirement for RRT on survival was analysed using a matched dataset and CPH model. Propensity-score based matching achieved excellent covariate balance (S3 Table) across the full range of the propensity score (S2 Fig). For those receiving RRT, risk of patient death at one year remained similar regardless of serum creatinine concentration (Fig 3A). However, an inverse relationship was seen between serum creatinine and graft survival in those not receiving RRT, while in the presence of RRT risk of graft failure remained similar (Fig 3B and S5 Table). Propensity-score based matching demonstrated that at a serum creatinine greater than 175 μmol/L, patients not receiving RRT had a significantly worse risk of graft failure than all those receiving RRT (Fig 3C). In total 56 patients (21.1%) had a serum creatinine greater than 175 μmol/L but did not receive RRT. In the unmatched dataset the result was similar, with a serum creatinine greater than 200 μmol/L in those not receiving RRT demonstrating a worse risk of graft failure (S3 Fig).

## Discussion

This is the first study to our knowledge to concurrently investigate the influence of renal replacement therapy (RRT) and pre-operative serum creatinine on survival following liver transplantation for acute liver failure. This retrospective analysis of a national population database demonstrated that RRT and serum creatinine were both independent predictors of patient survival, while creatinine was also a predictor of graft loss. For those not on RRT, outcome gets worse as creatinine rises. Even in patients with only a moderately elevated creatinine, outcomes were equivalently poor to those receiving RRT. When accounting for potential confounding factors, elevated serum creatinine predicted an increased risk of graft failure for those not receiving RRT, however this effect was lost in the presence of RRT.

Half of the cohort in this study required RRT prior to transplantation for acute liver failure, which is consistent with published literature [6]. Elevated serum creatinine and use of RRT are likely to represent an acute deterioration in renal function in this cohort and previous studies demonstrate a clear association between ALF and acute kidney injury [5]. Renal dysfunction is known to independently predict patient and graft survival in liver transplantation for any indication [3, 11], with the increased sepsis rate associated with renal dysfunction hypothesised as a possible explanation [7–9]. In acute liver injury, our results suggest that renal dysfunction is a measure of progressive organ system failure. This is supported by our observation of the additive negative effect of mechanical ventilation on patient survival and that renal dysfunction is known to prolong intensive care unit stay [4, 7].

In acute liver failure, a requirement for RRT may be a surrogate for metabolic dysfunction or systemic complications. Factors such as disseminated intravascular coagulation, hypovolaemia and sepsis all increase the likelihood of renal dysfunction [19, 20]. Our methods attempted to control for such confounding, with RRT associated with worse survival, independent of biochemical/coagulation disturbance and the requirement for mechanical ventilation, variables likely to reflect critically unwell patients. RRT is associated with poor outcomes in liver transplantation for indications other than acute liver failure [21]. It has been suggested that RRT in liver transplant recipients increases the risk of both bacterial and fungal sepsis [22, 23]. We found a greater proportion of deaths in patients with RRT attributed to sepsis (15 vs. 7), however the numbers here are too small to draw firm conclusions.

RRT has the ability to correct electrolyte imbalance, acid-base disturbance and limit coagulopathy [24, 25]. The function of RRT to correct physiological parameters could explain the effect of RRT on graft survival in a propensity-score matched data set. For example, it is well described that lactic acidosis is associated with increased hepatocellular damage and necrosis in the immediate period following liver transplantation [26, 27]. Correction of metabolic acidosis through the use of RRT may reduce hepatocellular damage and as a consequence minimise risk of graft dysfunction.

In contrast we did not demonstrate the presence of pre-operative RRT significantly reduced patient mortality following liver transplantation for ALF. This finding has been replicated previously in observational studies [28]. Other pre- and post-operative factors are likely to contribute to patient survival, with evidence suggesting response to various therapeutic strategies at day 7 post-transplantation (such as oxygen saturation, hypotension and level of inotropic support) predict survival [29, 30]. This suggests delayed recovery from multi-organ dysfunction following transplantation is an important marker for survival and therefore the cumulative effect of multi-organ dysfunction is likely to predict patient survival. As a result, the presence of RRT may have an additive effect on patient mortality in the presence of other organ support systems but may explain why it does not independently predict patient mortality. However, evidence suggests that the same patient cohort also has a higher rate of acute rejection and hence graft failure [28]. RRT may therefore provide an important, currently unidentified, individual mechanism in reducing graft failure compared to other organ support systems (as discussed previously) that would explain the effect of RRT in improving graft survival in this study.

Clinical studies suggest that increased doses of haemofiltration are associated with a reduced mortality in patients with sepsis [31, 32], possibly explained by increased removal of sepsis mediators from the circulation [33]. However controlled trials have demonstrated higher intensity RRT has no benefit, either on survival or renal recovery, in patients with acute kidney injury secondary to sepsis [34, 35]. Furthermore, a multi-centre randomized trial suggested early application of venovenous hemofiltration in a similar cohort of patients was deleterious to organ function [36]. Studies investigating intensity and timing of RRT in patients requiring transplantation for ALF are yet to be performed. Further work requires to be done on identifying the optimum timing for commencement of RRT, but commencing RRT early or using a low threshold for its institution may turn out to be beneficial.

Currently, our study demonstrates the potential of RRT in improving graft survival if commenced when a patient’s pre-operative creatinine rises above 175 μmol/L. The authors suggest that further prospective evidence is required before firm conclusions can be made regarding its implication in clinical practice, however the commencement of RRT should be considered in those patients where creatinine rises above 200 μmol/L. In addition, a creatinine of <200 μmol/L would not necessarily be contraindicated if it is required for other purposes such as refractory pulmonary oedema or hyperkalaemia.

The retrospective and observational nature of this study has limitations. Unfortunately data were not available for patient baseline renal function prior to development of acute liver failure, and despite attempting to account for confounding factors with propensity-score matching, an element of confounding may remain in the form of RRT indication, timing and modality, which is known to be diverse in clinical practice [35]. Furthermore pre- and post-operative Sequential Organ Failure Assessment (SOFA) and Model for End-Stage Liver Disease (MELD) scores, known to accurately predict short-term prognosis in liver transplantation [28], are not available within the dataset. This may limit the generalization of our findings. Further investigation of RRT in patients with ALF would be useful, particularly whether early initiation of RRT has a beneficial effect.

This study has important implications in relation to the management patients with renal impairment prior to transplantation. We have demonstrated that RRT is a predictor of patient death, while serum creatinine concentration independently predicts graft failure in the absence of RRT. By demonstrating graft survival is equivalent in those patients receiving RRT regardless of serum creatinine concentration, further studies need to identify whether timing and indication of pre-operative RRT improves long-term graft survival in those undergoing liver transplantation for acute liver failure.

## Supporting Information

S1 Table UK Transplant Super Urgent Scheme Categories. (TIFF)

S2 Table Life style activity score codes. (TIFF)

S3 Table Covariate balance between treatment (renal replacement therapy) and control (no renal replacement therapy) groups following full matching propensity-scoring. (TIFF)

S4 Table Comparison of renal replacement therapy requirement and aetiology of graft failure and patient mortality following liver transplantation for acute liver failure. (TIFF)

S5 Table Cox Proportional Hazards model after full propensity-score matching. (TIFF)

S6 Table Exploring the effect of UK Transplant centre using a frailty/random effect in the Cox Proportional Hazard models on patient and graft survival. (TIFF)

S1 Fig Calibration curve for final Cox proportional hazards model. Bootstrapping was used to get bias-corrected estimates of predicted versus observed values. Predicted survival probability at 1 year is shown against observed survival (Kaplain-Meier survival estimate). Histogram at top of figure represents actual numbers of patients with these survival times. X, re-sampling optimism added (number of repetitions = 40). Error bars are 95% confidence intervals for Kaplan-Meier estimates.
(TIFF)

S2 Fig Distribution of propensity scores using “full matching” technique in treated (renal replacement therapy) and control (no renal replacement therapy) groups. Matching performed using all available recipient variables: gender, age, ethnicity, cause of liver failure, life-style activity score, ventilation status, encephalopathy grade, intracranial pressure monitoring status, sepsis status, haemoglobin, white cell count, platelet count, albumin, bilirubin and sodium.
(TIFF)

S3 Fig The effect of renal replacement therapy on graft survival in an unmatched dataset. In patients not receiving RRT, a pre-operative serum creatinine of greater than 200 μmol/L had a significantly greater risk of graft failure compared to those receiving RRT (black line indicates hazard ratio = 1, shaded areas represent 95% CI). Multivariate Cox proportional hazards model using pre-operative risk factors contained in Table 3.
(TIFF)

## Abbreviations

ALF: Acute liver failure
CPH: Cox proportional hazards
IQR: Interquartile range
NHS: National Health Service
RRT: Renal replacement therapy
