# Supplementary figures and images for: Use of Renal Replacement Therapy May Influence Graft Outcomes following Liver Transplantation for Acute Liver Failure: A Propensity-Score Matched Population-Based Retrospective Cohort Study
Source: PLoS One. 2016 Mar 1;11(3):e0148782. doi: 10.1371/journal.pone.0148782 (PMC4773220; doi:10.1371/journal.pone.0148782)

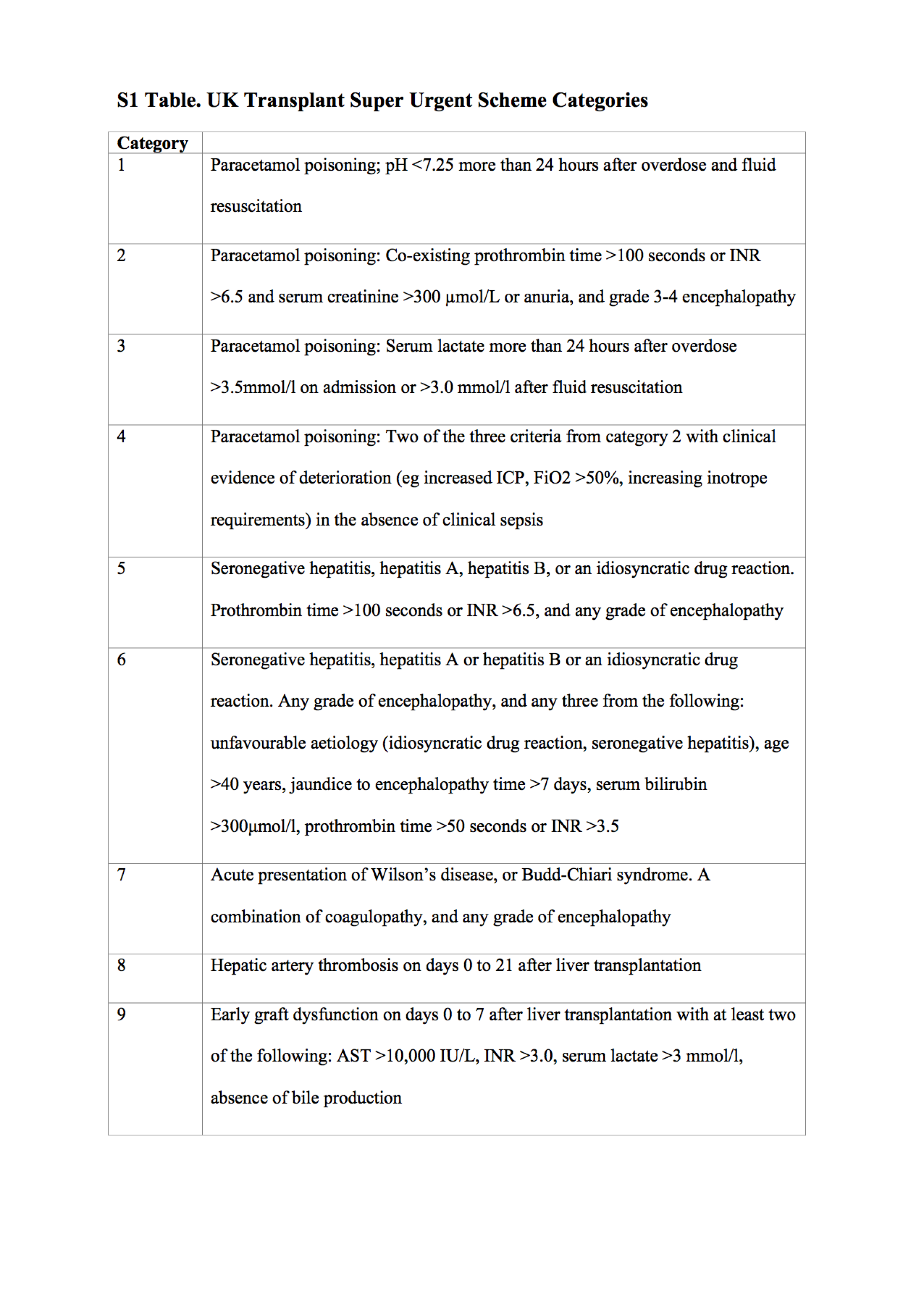

Supplement: S1 Table — (TIFF) [file pone.0148782.s001.tiff]

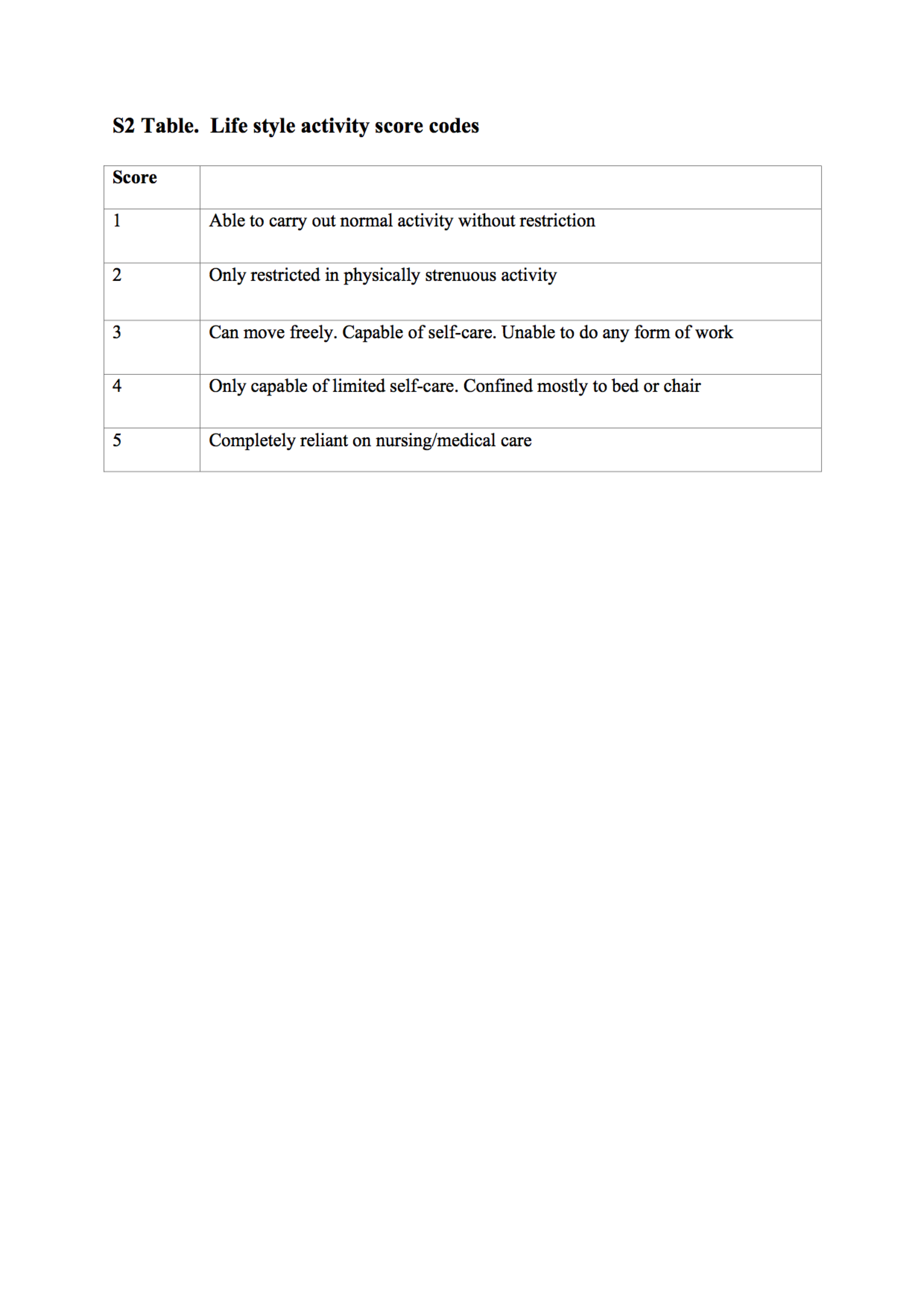

Supplement: S2 Table — (TIFF) [file pone.0148782.s002.tiff]

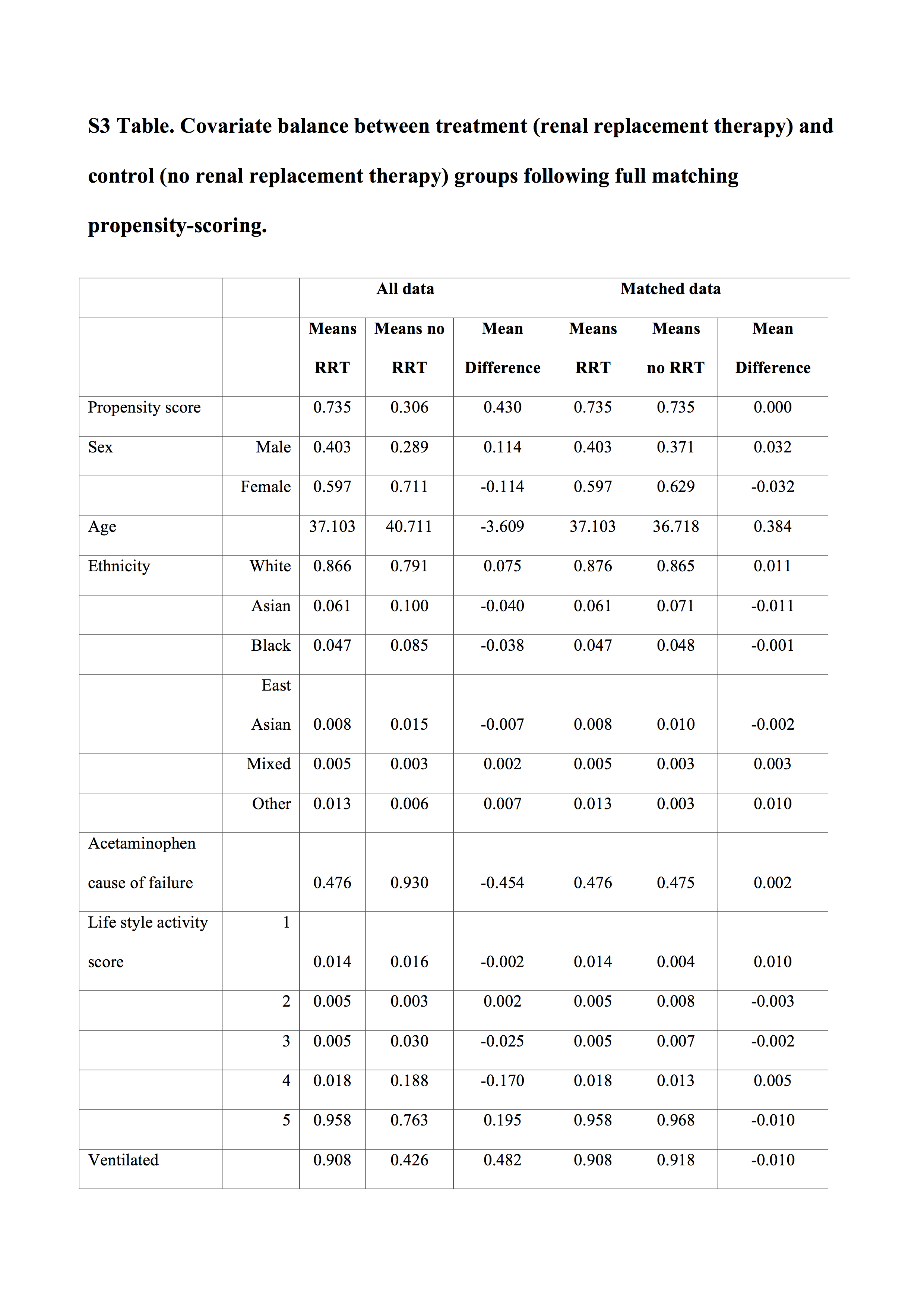

Supplement: S3 Table — (TIFF) [file pone.0148782.s003.tiff]

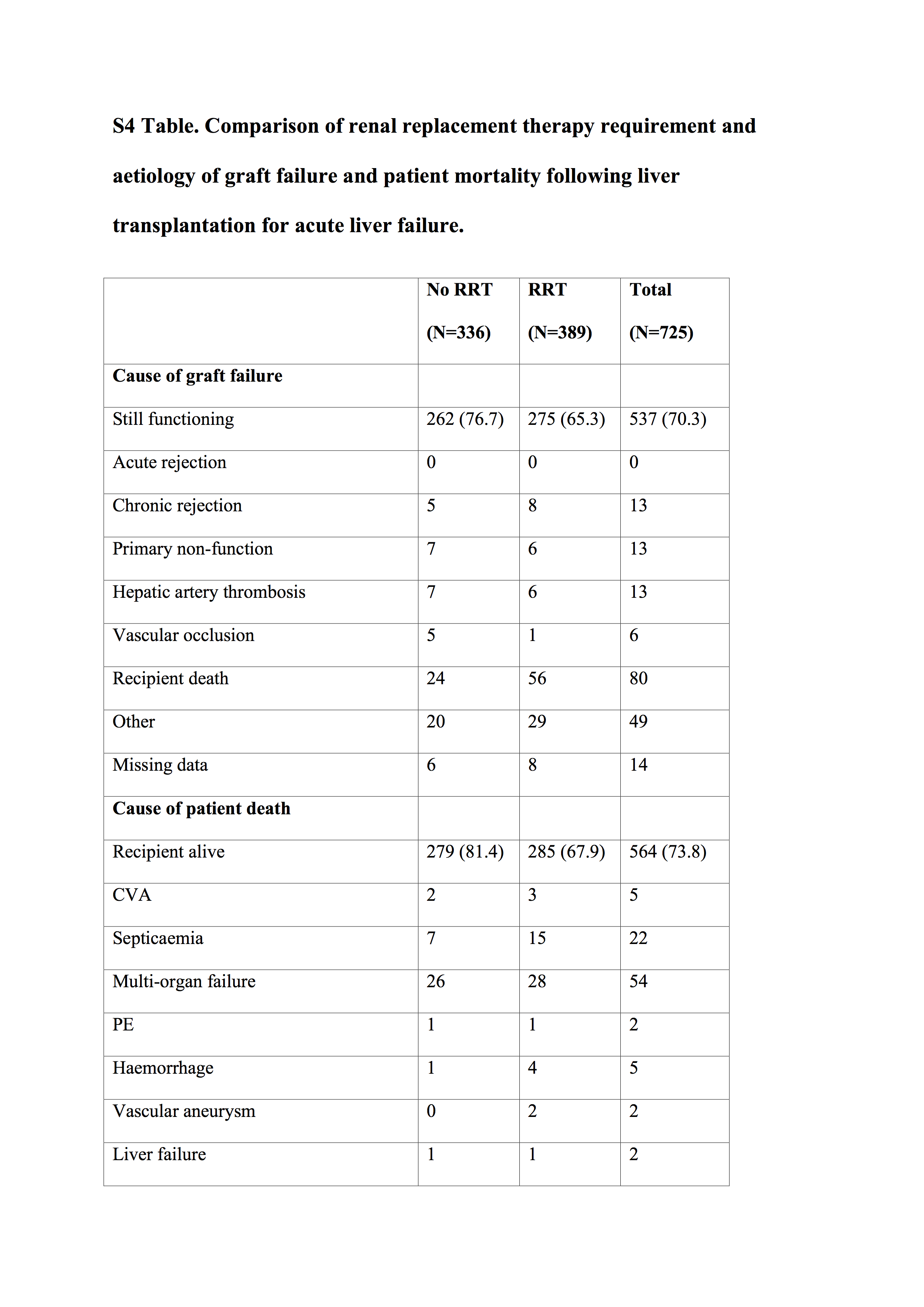

Supplement: S4 Table — (TIFF) [file pone.0148782.s004.tiff]

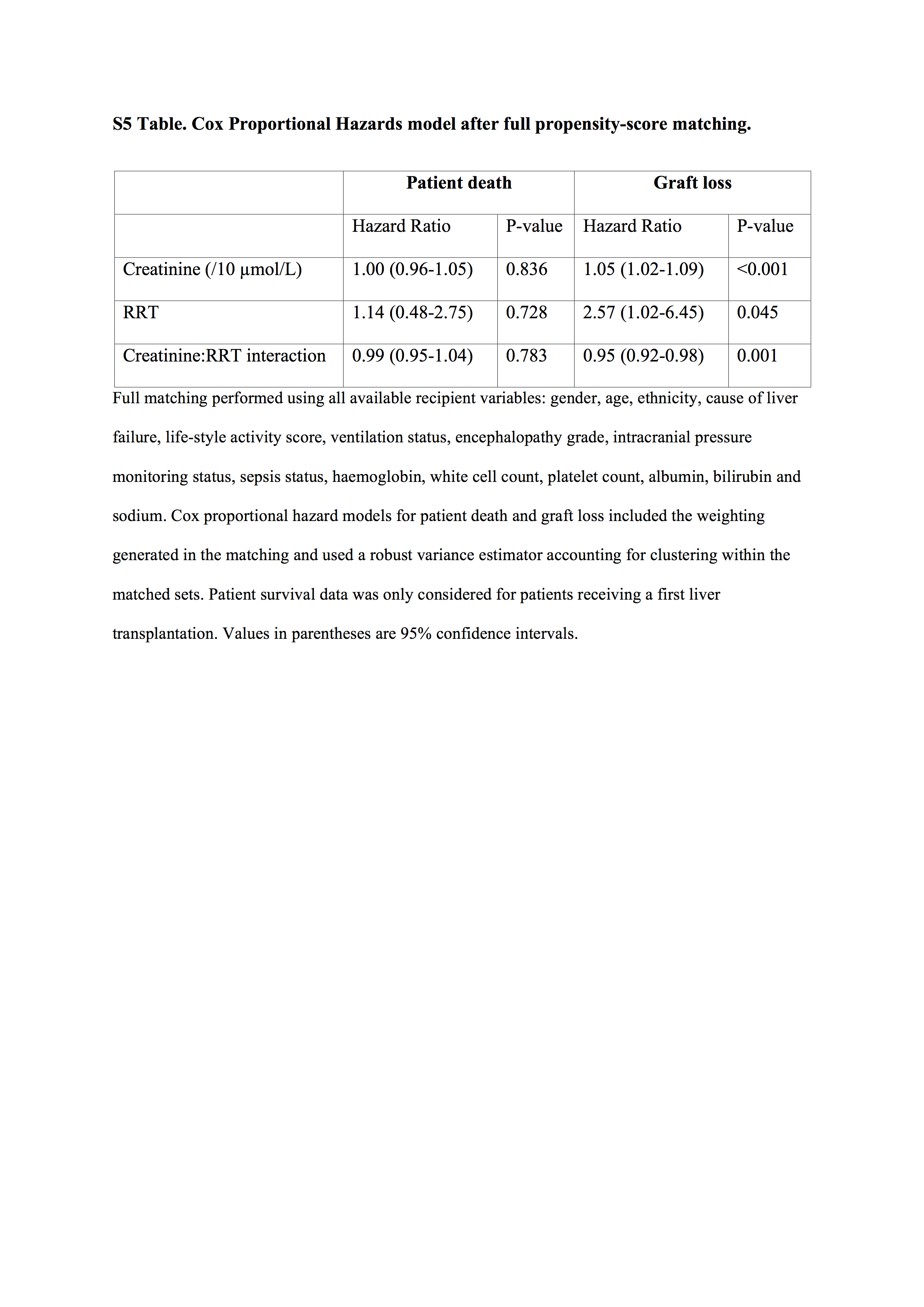

Supplement: S5 Table — (TIFF) [file pone.0148782.s005.tiff]

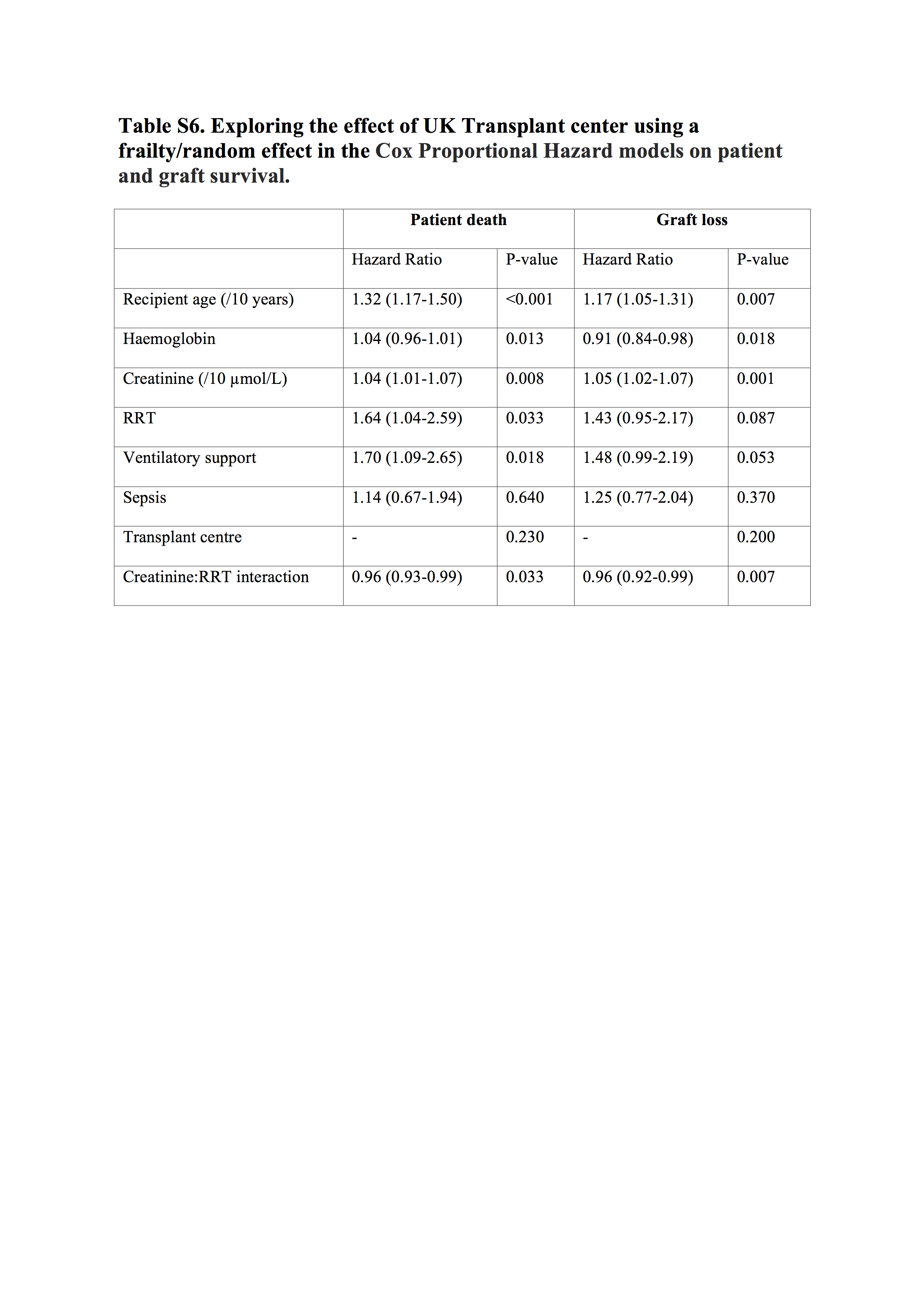

Supplement: S6 Table — (TIFF) [file pone.0148782.s006.tiff]

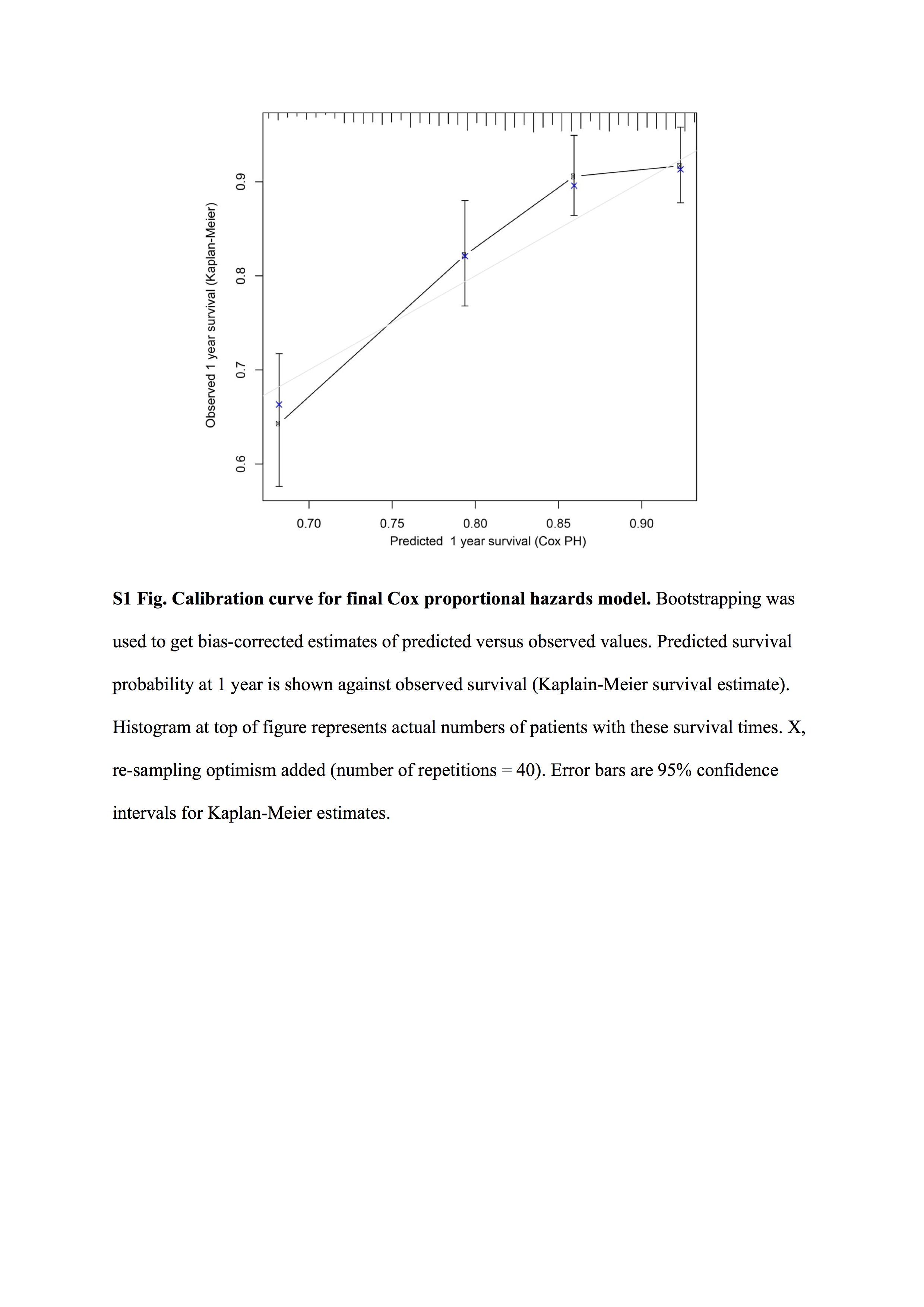

Supplement: S1 Fig — Bootstrapping was used to get bias-corrected estimates of predicted versus observed values. Predicted survival probability at 1 year is shown against observed survival (Kaplain-Meier survival estimate). Histogram at top of figure represents actual numbers of patients with these survival times. X, re-sampling optimism added (number of repetitions = 40). Error bars are 95% confidence intervals for Kaplan-Meier estimates. (TIFF) [file pone.0148782.s007.tiff]

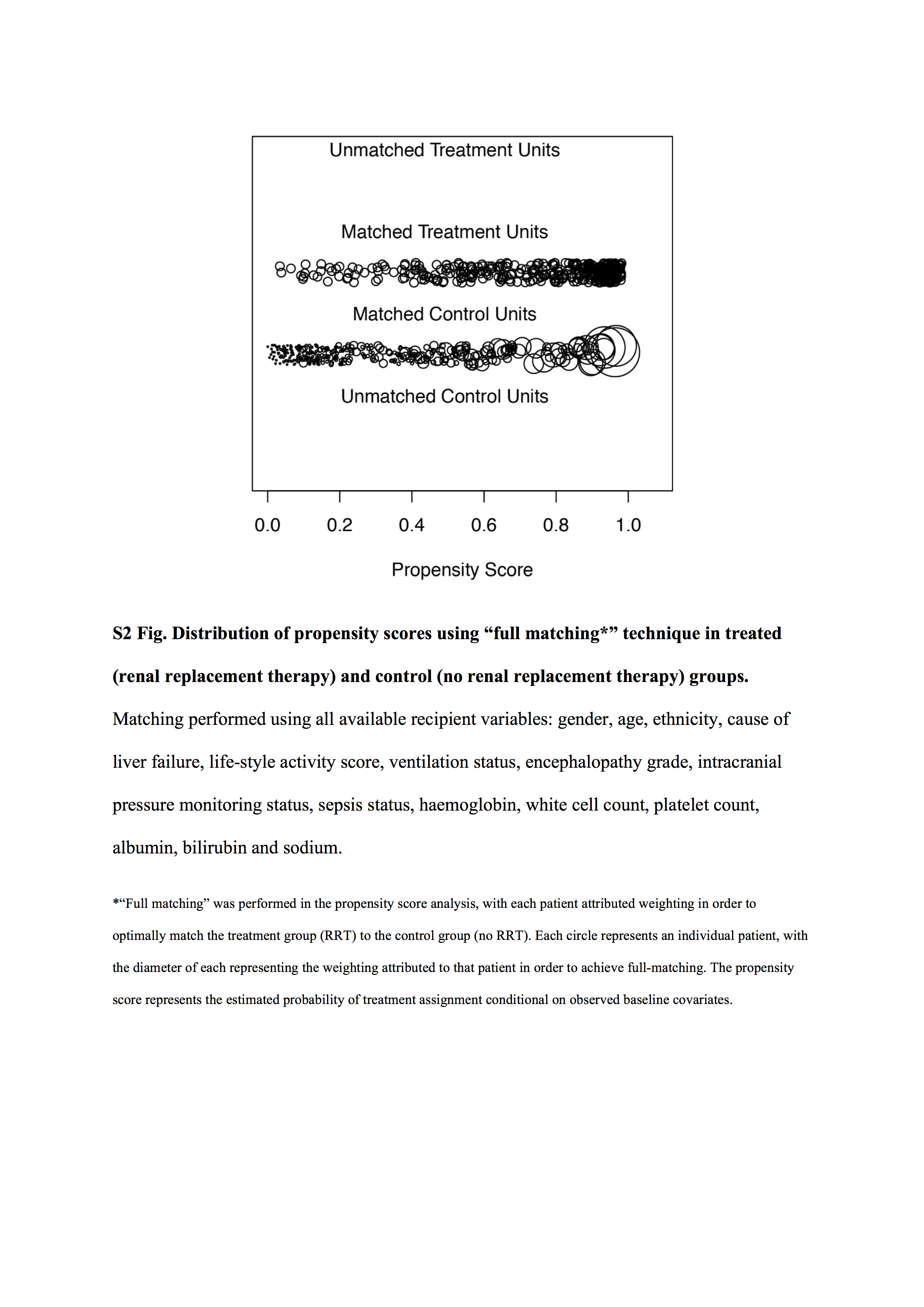

Supplement: S2 Fig — Matching performed using all available recipient variables: gender, age, ethnicity, cause of liver failure, life-style activity score, ventilation status, encephalopathy grade, intracranial pressure monitoring status, sepsis status, haemoglobin, white cell count, platelet count, albumin, bilirubin and sodium. (TIFF) [file pone.0148782.s008.tiff]

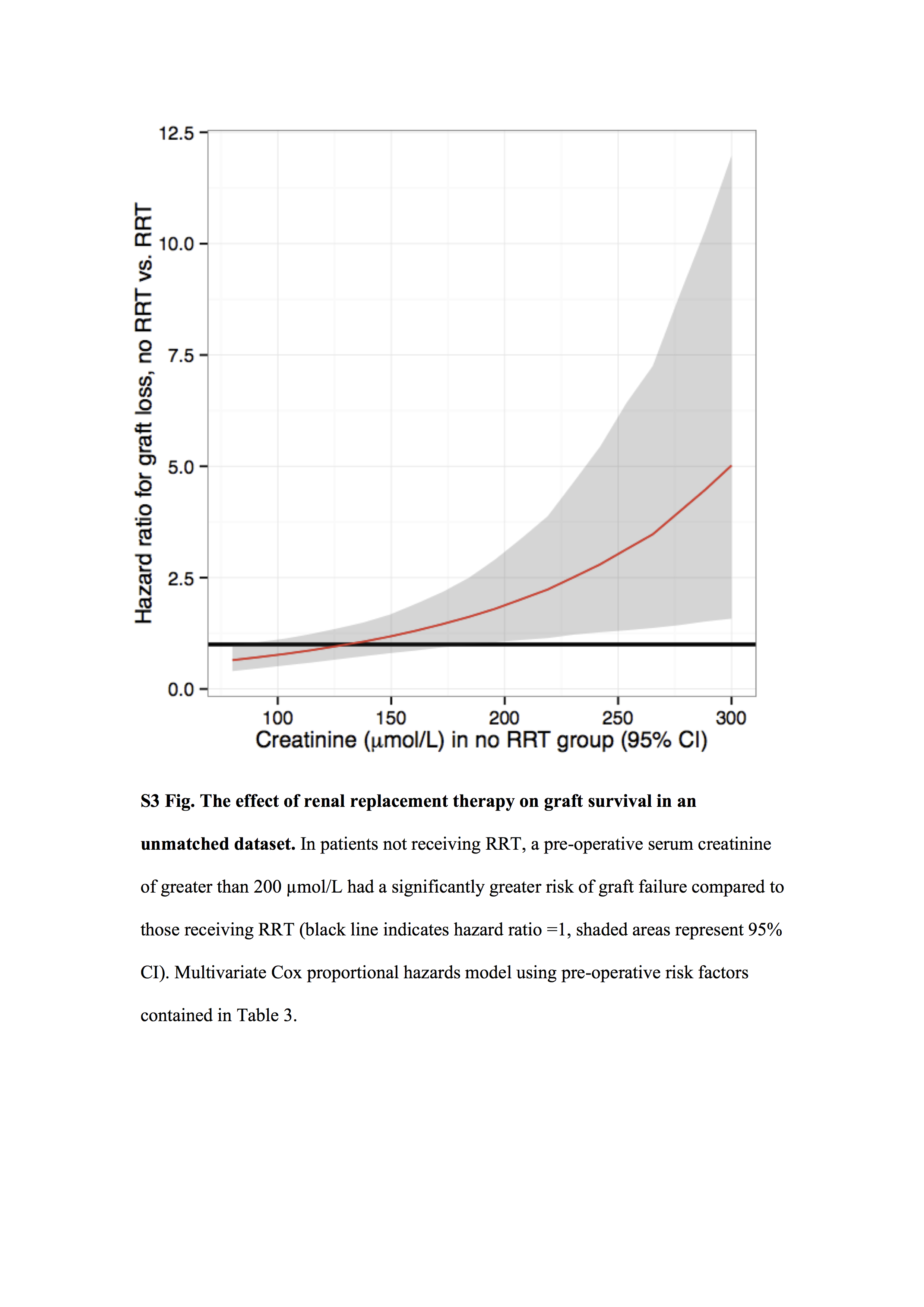

Supplement: S3 Fig — In patients not receiving RRT, a pre-operative serum creatinine of greater than 200 μmol/L had a significantly greater risk of graft failure compared to those receiving RRT (black line indicates hazard ratio = 1, shaded areas represent 95% CI). Multivariate Cox proportional hazards model using pre-operative risk factors contained in Table 3. (TIFF) [file pone.0148782.s009.tiff]
